# Sarcopenia screening strategies in older people: a cost effectiveness analysis in Iran

**DOI:** 10.1186/s12889-021-10511-7

**Published:** 2021-05-17

**Authors:** Ali Darvishi, Mohsen Rezaei Hemami, Gita Shafiee, Rajabali Daroudi, Mahsa Mohseni, Farkhondeh Hosseini Shekarabi, Ramin Heshmat

**Affiliations:** 1 Chronic Diseases Research Center, Endocrinology and Metabolism Population Sciences Institute, Tehran University of Medical Sciences (TUMS), NO 10, Jalale-Al-Ahmad Ave, Chamran Highway, Tehran, 1411713137 Iran; 2 Department of Health Management and Economics, School of Public Health, Tehran University of Medical Sciences, Poursina AVE, Tehran, 1417613151 Iran; 3 Aberdeen Centre for Health Data Sciences, University of Aberdeen, Foresthill, Aberdeen, AB252ZD UK; 4 National Center for Health Insurance Research, Iran Health Insurance Organization, Tehran, Iran; 5 Department of Mathematics, Faculty of Sciences, Shahid Rajaee Teacher Training University, Lavizan, Tehran, 1417613363 Iran

**Keywords:** Sarcopenia, Screening, Cost effectiveness analysis (CEA), QALY

## Abstract

**Background and objectives:**

Sarcopenia is an important age-related disease which can lead to an increased risk of mortality, falls, fractures, and poor quality of life. So, timely detection can be effective in reducing the burden of disease. The aim of this study was to identify the most cost-effective strategy for sarcopenia screening in Iran.

**Materials and methods:**

We constructed a Markov transition model over a life-time horizon based on natural history. Compared strategies included Sarcopenia scoring assessment models (SarSA-Mod), European working group on sarcopenia in older people (EWGSOP), Mini sarcopenia risk assessment (MSRA) and SARC-F. Parameters values were extracted from primary data and the literature, and the costs and Quality-adjusted life years (QALYs) were calculated for each strategy. Sensitivity analysis of uncertain parameters was also performed to determine the robustness of the model. Analysis was performed using 2020 version of TreeAge Pro software.

**Results:**

All four screening strategies increased life time QALYs. After removing dominated strategy, the incremental cost per QALY gained for sarcopenia screening varied from $1875.67 for EWGSOP to $1898.33 for MSRA. Our base-case analysis showed that the most cost-effective strategy was EWGSOP and 2nd best was SarSA-Mod with $43,414.3 and $42,663.3 net monetary benefits given one GDP per capita ($5520.311) as willingness to pay, respectively. Sensitivity analysis of model parameters also showed robustness of results.

**Conclusions:**

The results of the study, as the first economic evaluation of sarcopenia screening, showed that the EWGSOP strategy is more cost-effective than other strategies.

**Supplementary Information:**

The online version contains supplementary material available at 10.1186/s12889-021-10511-7.

## Introduction

One of the important topics in geriatric medicine is sarcopenia or loss of age-related skeletal muscles that has been considered more in recent years [1, 2]. Sarcopenia is defined as syndrome with the loss of progressive skeletal muscle mass and function that is significantly accompanied with increasing mortality, reducing quality of life, and increasing costs of the elderly care [3, 4]. During the years of 50 to 80 of life, sarcopenia reduces muscle mass by about 30% [4] and is accompanied with myasthenia, increasing fatigue and inappropriate function in elderly which may cause to reduce individual’s ability in moving quickly and increase the risk of falling [5, 6].

Sarcopenia can harms on society health and welfare and on the other hand it leads to increase the health costs, the risk of physical disability, reducing the quality of life, increase the need for care services, and ultimately increase the risk of mortality in elderly age groups. Therefore, in recent decades it has attracted the attention of researchers around the world [4, 6–8]. Impotence and dependency in personal affairs, falling and fracture are the most important complications that have led to consider osteoporosis and sarcopenia as a movement disorder syndrome [9]. In addition, the results of studies have shown that sarcopenia is associated with other diseases such as metabolic syndrome, respiratory diseases as well as cardiovascular diseases (CVDs) [10–12]. Considering all the different aspects of the disease, sarcopenia can have a huge economic burden on the health care system and society welfare.

It is estimated that sarcopenia occurs in 5 to 45% of the elderly population [13–16]. Although sarcopenia occurs mainly in the elderly, there are also cases in younger people. A study conducted by Cherin revealed that 9% of individuals between the ages of 45 to 54 years and 13.5% of people aged 55 to 64 are sarcopenic [17]. Other studies have shown that the rate of sarcopenia is very common in elderly hospitalized patients and is about 10 to 37.3%, of which about one-fifth of these patients are under 65 years old [18–21]. The results of sarcopenia frequency assessment in Iran based on the definition of the EWGSOP for 300 people of over the age of 55 years showed that 30% of men and 18% of women are sarcopenic [22–24]. The prevalence of sarcopenia is related to the type of muscle mass measuring instrument [2]. Available instruments include bioimpedance analysis (BIA) and Dual-energy X-ray absorptiometry (DXA) which have different measurement accuracy and cost. A review of studies shows that in general, the prevalence of sarcopenia based on BIA instrument is estimated higher than DXA and in other words the prevalence is overestimated by using the BIA instrument [2].

Prevalence of the sarcopenia is high in Iran, as well as complications and consequences of this disease is considerable, so screening at-risk individuals and also performing effective interventions at the beginning of the disease progression could have a significant impact on reducing the negative consequences of this disease. Therefore, in the early stages of the disease, that the functional disorder has not yet occurred, there may be a good opportunity to perform interventions such as supplements, nutritional diet and exercise to reduce the progression of the disease [25–27].

Screening strategies and instruments are used in the early detection of at risk people for sarcopenia in the clinical and research section. Overall, existing screening strategies are screening questionnaire, diagnostic algorithms, or predictive equations [2]. There is a wide range of sarcopenia measurement components including body muscle mass and muscle strength and performance whereas the most important symptom of sarcopenia is reducing skeletal muscle mass [2]. Different methods are vary in accuracy, cost, and duration of measurement. In many countries, it is not possible to perform these tests especially examining the muscle mass measurement by DXA. Therefore, researchers seek easier ways for assessing and screening of sarcopenia [2]. The first screening strategy was presented by EWGSOP in 2010, which is a two-step algorithm [28]. The Asian working group for sarcopenia (AWGS) also provided an algorithm in order to screen sarcopenia in the elderly people in 2014 [2]. A questionnaire called SARC-F was also designed by Malmstrom and Morley, which consists of five sections. This questionnaire can detect older people who need to additional assessment for sarcopenia (high-risk individuals) [29]. Ishii and colleagues (2014) innovated another method for detection of the elderly people at risk of sarcopenia that can estimate possibility of sarcopenia [26]. Yu and his colleagues in Australia, using a predictive equation of the anthropometry components with muscle strength, can detect people who are at risk of sarcopenia [30]. Mini sarcopenia risk assessment (MSRA) questionnaire is also designed by Rossi et al. which can detect at risk people [31]. A model called sarcopenia scoring assessment model (SarSA-Mod) has been recently designed in Iran that can help to detect people at risk of sarcopenia with high accuracy [32].

The existing information on economic burden of sarcopenia is limited and not many studies have been conducted on this subject. A study, that is conducted on a sample of people over than 60 years old in USA in 2004, has shown that the health care cost related to sarcopenia is about $ 18.5 billion ($ 10.8 billion for men and $ 7.7 billion for women) [33]. Considering population aging as well as the health and economic side effects of the disease progression and limitation of health care financial sources, so the expanding the screening for sarcopenic people and early detection of at-risk individuals and administering timely interventions are very important. On the other hand, considering that there are different screening methods with various costs and effectiveness, the present study aims to determine the cost and effectiveness of sarcopenia screening strategies in Iran and tries to provide the evidence and information needed for policy and decision making for applying best strategy in this regard.

## Materials and methods

The present study is a full economic evaluation which was made a comparison of four sarcopenia screening strategies as well as no screening. Study was conducted in two phases. In order to review available evidences in this subject, in first phase a systematic review study of economic evaluation studies for sarcopenia screening was conducted to examine how to evaluate parameters and challenges ahead and use of its results in designing economic evaluation model. However, economic evaluation study related to sarcopenia was not found based on our inclusion criteria (systematic review results of the first phase of the study could be seen in the form of the Prisma flow diagram in the supplementary information (Fig. S1). Therefore, in phase two of the study, for the first time, we developed a cost-effectiveness analysis model to compare sarcopenia screening strategies.

### Modeling

Designing cost-effectiveness analysis model was conducted based on the natural history of disease, how and the protocol of performing the four screening methods, the clinical outcomes, the probabilities of the occurrence of the consequences and the occurrence of the costs. In order to design the model, specialized panels were formed with the participation of the study team of clinical experts and the economic team. After the trial and error of different models, the final model was extracted with consensus and considering the most important clinical and economic outcomes based on the diagnosis and treatment of disease natural history.

According to the review of the clinical evidence of the disease and also the results of the expert opinions, four variables were finally considered as the intermediate outcome. Increasing the risk of falling and consequently the risk of fracture, increasing the risk of CVDs due to sarcopenia, increasing the risk of death and reducing the quality of life were considered as the main outcome of the model. Comorbidities have also been omitted due to the simplification of the model and the ability to evaluate.

**Fig. 1:**
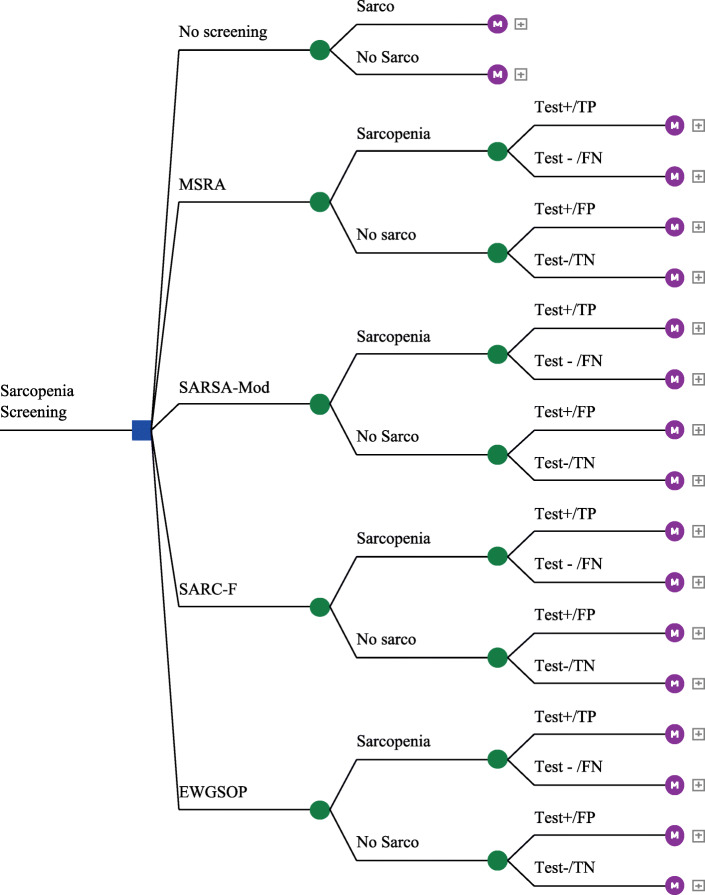
Decision Tree Model for CEA of Sarcopenia Screening Strategies

Structure of the model was designed in two parts: the decision tree and markov transition model. Decision tree is developed based on the evidence and process of screening tests. In this model, individuals enter to decision tree and the process of events is considered the same in each of the strategies. After entering the model, individuals are divided into two groups according to the prevalence of sarcopenia: sarcopenic and healthy. In each of the two groups, the test results could be positive or negative (false and true positives or false and true negatives) (Fig. 1). Then all the groups enter the Markov model depending on whether they are sarcopenic or healthy.

### Markov model

**Fig. 2:**
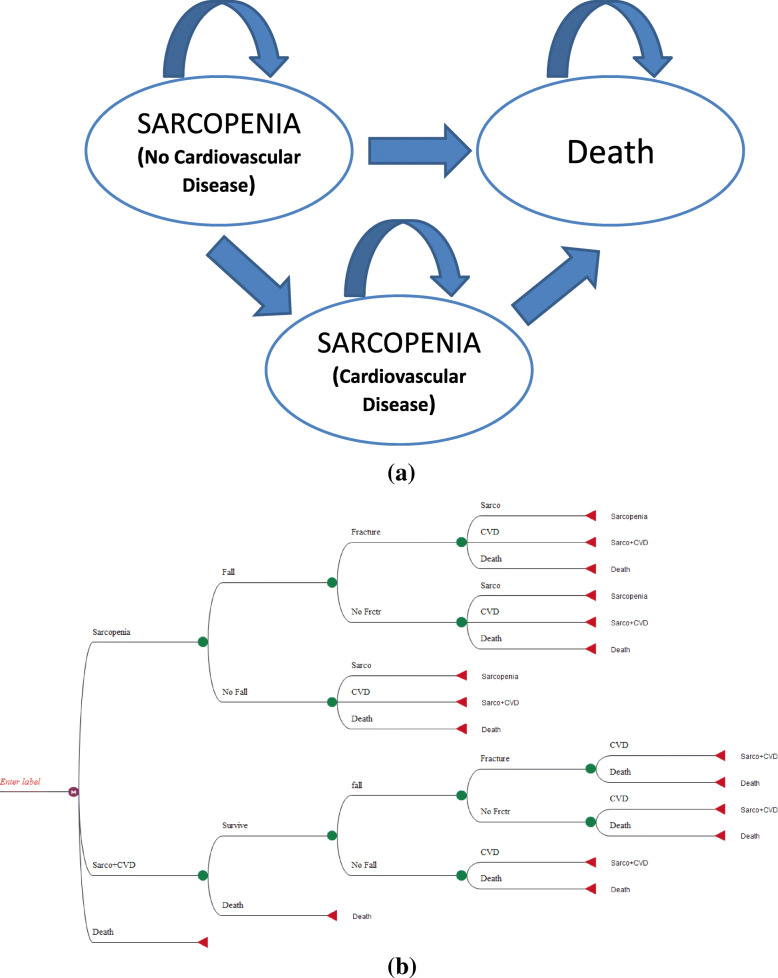
Markov Model for CEA of Sarcopenia Screening Strategies

The schematic and simple structure of the Markov model used in this study can be seen in Fig. 2. We considered 1000 study populations in the hypothetical Markov cohort.

The model consists of three health states: sarcopenia without cardiovascular disease, sarcopenia with cardiovascular disease and death, and individuals are in transition during the simulation and study period according to the probability of transition between these conditions so that at the end of each cycle individuals remain in the same health state or are transferred to other health state, and eventually in the final cycle all individuals transferred to death health state.

### Model assumptions

Our model has the following rules and assumptions:

Each strategy, regardless of its stages, acts as a single test, which ultimately has a positive or negative response, which is classified as true and false positives and true and false negatives.

The progression of the disease in Markov’s structure is forward-looking for more simplicity and is considered irreversible.

In this model, the effectiveness of timely diagnosis and treatment is considered in the probability of transitions between states as well as the risk of occurrence of outcomes such as the risk of falls and fractures.

In false positive cases, patients are examined clinically and undergo a therapeutic intervention cycle, and this is the only additional cost that is imposed on this group of patients.

Markov’s time horizon, with regard to the life expectancy of Iranian men and women aged 60 years, was considered to be 25 years. The length of Markov cycles is also considered to be 1 year due to the nature of the disease state of changes. Since the individuals could survive less than 1 year, we apply the half-cycle correction to adjust for the potential overestimation of the costs and utilities.

It should be noted that sarcopenic patients are usually divided into three groups: Pre-sarcopenia, sarcopenia and severe sarcopenia [34], but in the current Markov structure, the patients are considered all in one group and the health state of the accumulated sarcopenia and the division of patients into three groups has been discarded due to changes in definitions of pre-sarcopenia in recent years and the disagreement over them and the lack of access to the separate evidence [35, 36].

In the present model, fall and fracture are considered an event in life cycles. Due to this, after fall and fracture people have a lower quality of life and also incur new treatment costs.

Moreover, in this model, regarding the available limitations, it is assumed that screening and diagnosis are performed only once, and screening is not performed during Markov cycles, and costs have not been considered in this regard.

### Screening strategies and descriptions

In present cost-effectiveness analysis, we compared four sarcopenia screening strategies with the no screening strategy. The selection of screening strategies was based on Iran health care setting and which are used for sarcopenia screening in Iran and a strategy has been added to this comparison as a new method for sarcopenia screening.

1. The European Working Group on Sarcopenia in Older People (EWGSOP) algorithm: This method is done in two parts. In the first part, the muscle performance test and handgrip strength test are performed, and in the second part, according to the defined cut of points for the at-risk individuals in the first part, the muscle mass measurement is preferably performed using DXA [28].
2. SARC-F method: This method is based on a questionnaire designed by Malmstrom and Morley, which includes 5 parts. This questionnaire can detect elderly people who need supplementary assessment for sarcopenia (high-risk individuals) [29].
3. Mini Sarcopenia Risk Assessment (MSRA) method: This method is also based on the MSRA questionnaire designed by Rossi and his colleagues that can identify high-risk individuals. The questionnaire includes seven items including age, level of physical activity, hospitalization, weight loss, and three dietary questions [31].
4. Sarcopenia Scoring Assessment Models (SarSA-Mod) method: A model called SarSA-Mod has been designed which can identify sarcopenia at risk individuals. In this model, it evaluates the variables of age, weight, and calf circumference for men and women. In brief, to develop a statistical screening model to identify patients with sarcopenia, the study sample was randomly divided into two parts; 67% of the cases, called development set, which were allocated to the development of the model, and 33% of the dataset, named validation set were allocated to validation of the model. Logistic regression analysis with stepwise approaches was applied in the development of the model. ß coefficient of each variable was used to calculate its index weight. The ability of the model to separate those with sarcopenia from those without sarcopenia was evaluated using receiver operating characteristic (ROC) curves and the area under the ROC curve. A suitable cut-off point was selected with regards to sensitivity and specificity. Then, sensitivity, specificity, positive and negative predictive values (PPV & NPV) and the accuracy of the scores were evaluated using the validation set, which was left aside so far and not engaged in the model development process. In order to select and validate the final criteria for our scoring model, the model was applied to the validation set, using ROC analyses [unpublished data].
5. No screening.

### Extracting parameter values and how to analyze model

Parameters and variables related to screening strategies such as sensitivity, specificity, sarcopenia prevalence, probability of transitions, risks of fall and fracture and other parameters were extracted from the literatures. In this regard, a separate each discrete search parameter was performed based on keywords and specific search strategy in scientific databases, and studies that had appropriate evidence were classified, and finally the best available evidence was extracted. In cases where there was no evidence for the desired parameter, the values ​​of the parameters were estimated using the experts’ opinion (panel including clinical specialists and economic team) (Table 1).

**Table 1:** Main input parameters of CEA model

Statistic variable	Base case	SD/(CI)	Distribution	Source
Sarcopenia prevalence in Iran	0.245	±0.08	Beta	[24]
Annual discount rate	0.05	(0.03–0.12)	Beta	
Time Horizon (years)	25			
*Sensitivity of screening test*s				
EWGSOP	1	–	Beta	[32] and Table S1
SarSA-Mod	0.806	(0.767–0.842)	Beta	[32] and Table S1
MSRA	0.804	±0.08	Beta	[31]
SARC-F	0.168	(0.135–0.206)	Beta	[32] and Table S1
*Specificity of screening tests*				
EWGSOP	0.84	(0.816–0.862)	Beta	[32] and Table S1
SarSA-Mod	0.788	(0.75–0.803)	Beta	[32] and Table S1
MSRA	0.604	(0.543–0.664)	Beta	[31]
SARC-F	0.866	(0.843–0.886)	Beta	[32] and Table S1
*Probability of transition*				
Probability of death in non-sarcopenic individuals (Normal Pop 60 yrs)	0.09		Beta	IRI Life Table
Probability of death in sarcopenic individuals	0.132	±0.013	Beta	[37, 38]
Probability of death in sarcopenic individuals with cardiovascular disease	0.198	±0.03	Beta	[37, 38] And Calibration
Probability of death for sarcopenic individuals with fracture	0.171	±0.02	Beta	[37, 38] And Experts opinion
Probability of falling in sarcopenic individuals	0.273	±0.03	Beta	[39–41]
Probability of fracture in sarcopenic individuals	0.40	±0.05	Beta	[37]
Probability of cardiovascular disease (CVDs) in sarcopenic individuals	0.27	±0.03	Beta	[12] And Experts opinion
*Costs($)*				
Annual average cost of treatment interventions (vitamin D, Supplements, exercise, diet)	1119.048	±119.047	Gamma	Survey and Calibration
Cost of usual gait speed test	0.714		Gamma	Survey and Calibration
Cost of handgrip strength test	1.309		Gamma	Survey and Calibration
Cost of DXA	42.857		Gamma	Survey and Calibration
Cost of MSRA	0.714		Gamma	Survey and Calibration
Cost of SARC-F	0.714		Gamma	Survey and Calibration
Cost of SARC-F (high-risk individuals)	44.880		Gamma	Survey and Calibration
Cost of SarSA-mod	1.785		Gamma	Survey and Calibration
EWGSOP (stage 1)	2.023		Gamma	Survey and Calibration
EWGSOP (stage 2)	42.857		Gamma	Survey and Calibration
Cost of treatment and care of fractures	3599.048	±695.074	Gamma	Survey and Calibration
The initial average per capita cost of treatment and care of cardiovascular patients	4149.286		Gamma	Survey and Calibration
The incremental average per capita cost of treatment and care of cardiovascular patients	1904.762	±190.476	Gamma	Survey and Calibration
*Utilities*				
Non-sarcopenic individuals (Normal Pop > 60 yrs)	0.76		Beta	[42, 43]
Sarcopenic individuals	0.68		Beta	[44]
Sarcopenic individuals with fracture	0.51		Beta	[40, 44, 45]
Sarcopenic individuals with cardiovascular disease	0.56		Beta	[44, 46]
Sarcopenic individuals with cardiovascular disease and fracture	0.42		Beta	[44–47]
*Efficacy of interventions*				
Efficacy of sarcopenia interventions (vitamin D, Supplements, exercise, diet)	40%	±15%	Beta	[48–50]

Detailed information about sensitivity and specificity values of screening strategies are presented in Table S1.

### Utility

The primary outcome of the study is the number of identified sarcopenia cases. Because the present study is a full economic evaluation study, the final outcome in this evaluation is considered the Quality Adjusted Life Years Index (QALYs) and each screening strategy are finally evaluated based on cost per QALY unit. Evidence of quality of life and utility in any health state has been extracted from international studies (Table 1).

### Cost

The study was conducted from the health system perspective, and the time horizon, as mentioned, was considered 25 years, and the costs were estimated accordingly.

The cost of each screening strategy, including the cost of screening and diagnostic tests, is calculated based on the cost unit used in each intervention. Gathered data from internal and external related evidence were used to do this. Additionally, the cost of treatment interventions for sarcopenia, the cost of treatment and care for fractures and the cost of treatment of cardiovascular diseases were calculated based on treatment protocols, the opinions of a specialized team and based on public tariffs of the Ministry of Health of Iran in 2019.

All Costs were converted from Iran Rial to the US Dollar at the official currency rate of 42,000 Rial per Dollar.

### Cost-effectiveness analysis

Finally, in order to analyze and assess the cost effectiveness strategy, the incremental cost-effectiveness ratio (ICER) was used. The formula for this index is as follows [51]:

$$ ICER={C}_1-{C}_2/{E}_1-{E}_2 $$

In this regard, C represents the cost of strategies and E indicates the value of effectiveness of the strategies.

After calculating the ICER, the strategy with the lowest ICER is introduced as the most cost effective strategy.

We also used the threshold value equal to the Islamic Republic of Iran (IRI) one GDP per capita ($5520.311) introduced by the WHO as the cost effectiveness threshold (Willingness to pay) to identify the most cost effectiveness strategy.

### Sensitivity analysis

With regard to the uncertainty about some of the parameters used in the model, the deterministic sensitivity analysis (DSA) and the probabilistic sensitivity analysis (PSA) of the base case results were conducted. In the present study, first, a one-way deterministic Sensitivity Analysis was performed using tornado diagram for all uncertain parameters including sarcopenia prevalence, sensitivity and specificity of tests, test costs, treatment of fracture cost, interventions’ cost was estimated. Since the deterministic sensitivity analysis cannot fully demonstrate all the uncertainties in the parameters used, the PSA was also used. Thus, considering the possible distribution of uncertain variables using Monte Carlo Simulation, cost effectiveness acceptability curve, incremental cost effectiveness scatter plot and other reports were extracted. Distributions used in PSA are shown in Table 1. In cases where no evidence was found for the distribution of the parameter, 10–15% of the mean parameter value was considered as the standard deviation and appropriate distribution was selected according to the type of variable.

Modeling and analysis of base case results, as well as all stages of sensitivity analyses were performed using the 2020 version of Tree Age software.

## Results

### Base case

The results of the base case analysis can be seen in Table 2.

**Table 2:** Results of base case cost-effectiveness analysis

Strategy	Cost($)	Incr Cost($)^a^	Eff^b^	Incr Eff^c^	ICER($)	Special
No screening	1455.07	0	7.40	0	0	Base line
SARC-F	1809.41	354.34	7.59	0.19	1897.84	
SARSA-Mod	3140.83	1685.76	8.30	0.90	1881.96	
MSRA	3151.27	1696.20	8.30	0.89	1898.33	
EWGSOP	3518.75	2063.68	8.50	1.10	1875.67	

^a^Incremental costs, ^b^Effectiveness, ^c^Incremental Effectiveness

In Table 2, screening strategies are arranged from the lowest to the highest life time average costs, respectively, and the incremental cost, incremental effectiveness, and incremental cost-effectiveness ratio (ICER) were also calculated. No Screening was considered as the reference strategy for calculation of ICER. The ICER of the EWGSOP strategy was the lowest ICER value among the 5 strategies equivalent of $1875.67 per QALY units.

Considering the cost-effectiveness threshold of $5520.311 (equal to Iran GDP per capita in 2017), Net Monetary Benefits of EWGSOP, SarSA-Mod, MSRA, SARC-F and No Screening strategies were estimated $43,414.3, $42,663.3, $42,640.6, $40,080.6 and $39,404.3 respectively, which shows that after EWGSOP, using SarSA-Mod strategy has the highest net monetary benefits.

**Fig. 3:**
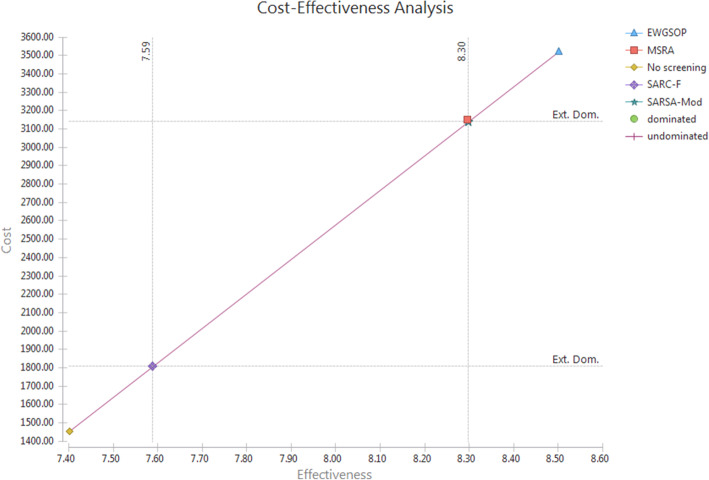
Cost-Effectiveness Plane of Sarcopenia Screening Strategies

Figure 3 shows the cost effectiveness plan of the study, which shows the placement of each of the strategies compared in this plot. Curve-based strategies are dominant and have lower cost and greater effectiveness (No screening, SARC-F, EWGSOP), and strategies that are outside of this curve are dominated strategies.

### Sensitivity analysis

Results of sensitivity analysis were performed in two sections: DSA and PSA which described separately.

#### Deterministic sensitivity analysis (DSA)

**Fig. 4:**
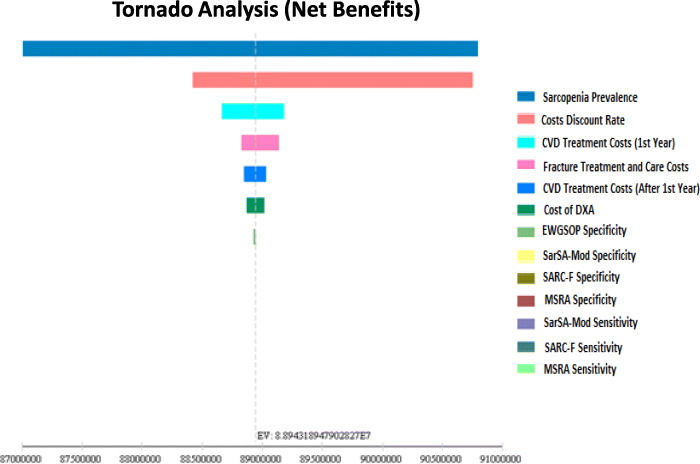
Deterministic Sensitivity Analysis using Tornado Diagram

In this section, the deterministic sensitive analysis was performed using one-way sensitivity analysis and tornado diagram. Accordingly, the rate of the sensitivity of the model results to uncertain parameters changes was measured. Sensitivity analysis results can be seen using a tornado diagram in Fig. 4. As shown, the results of the study are most sensitive to changes in the parameters of the sarcopenia prevalence and discount rate of sarcopenia treatment, CVDs and fracture costs.

It should be noted that although the changes in these parameters have changed the results of the model, the overall results of cost-effectiveness is not sensitive to changes in any of the uncertain parameters, in other words, these parameters do not have a threshold.

In general, the results of deterministic sensitivity analysis showed that the results of the base case are robust.

### Probabilistic sensitivity analysis (PSA)

PSA was performed using Monte Carlo Simulation regarding 1000 times of sampling replications.

**Fig. 5:**
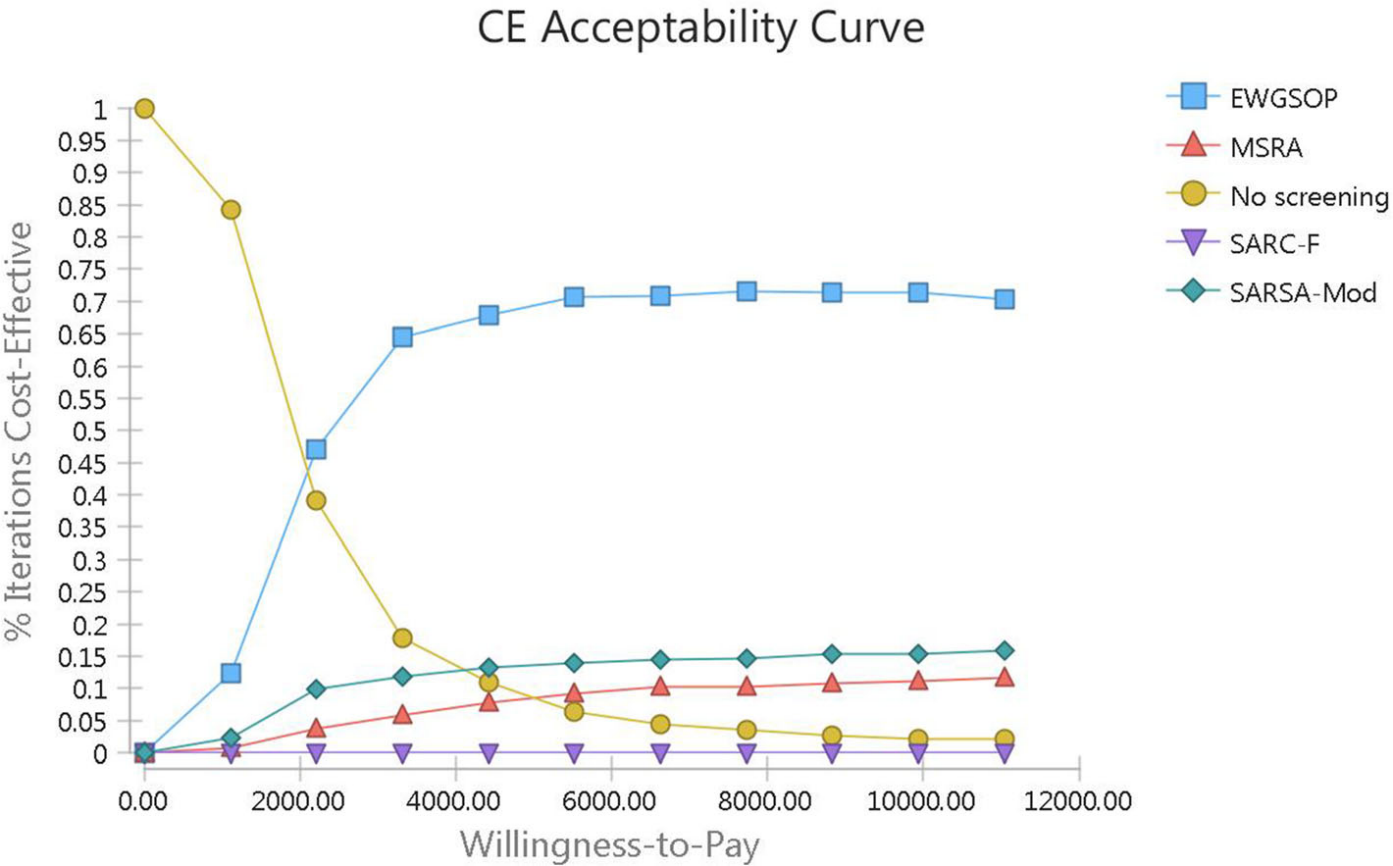
Cost Effectiveness Acceptability Curve of Sarcopenia Screening Strategies

Accordingly, the cost effectiveness acceptability curve (CEAC) was extracted (Fig. 5). As can be seen, increasing the threshold value increases the probability that the EWGSOP strategy will be cost effective and it reduces the probability of cost-effectiveness of the non-screening strategy. In addition, the probability of cost-effectiveness of other strategies in all threshold values ​​is about less than 20%.

**Fig. 6:**
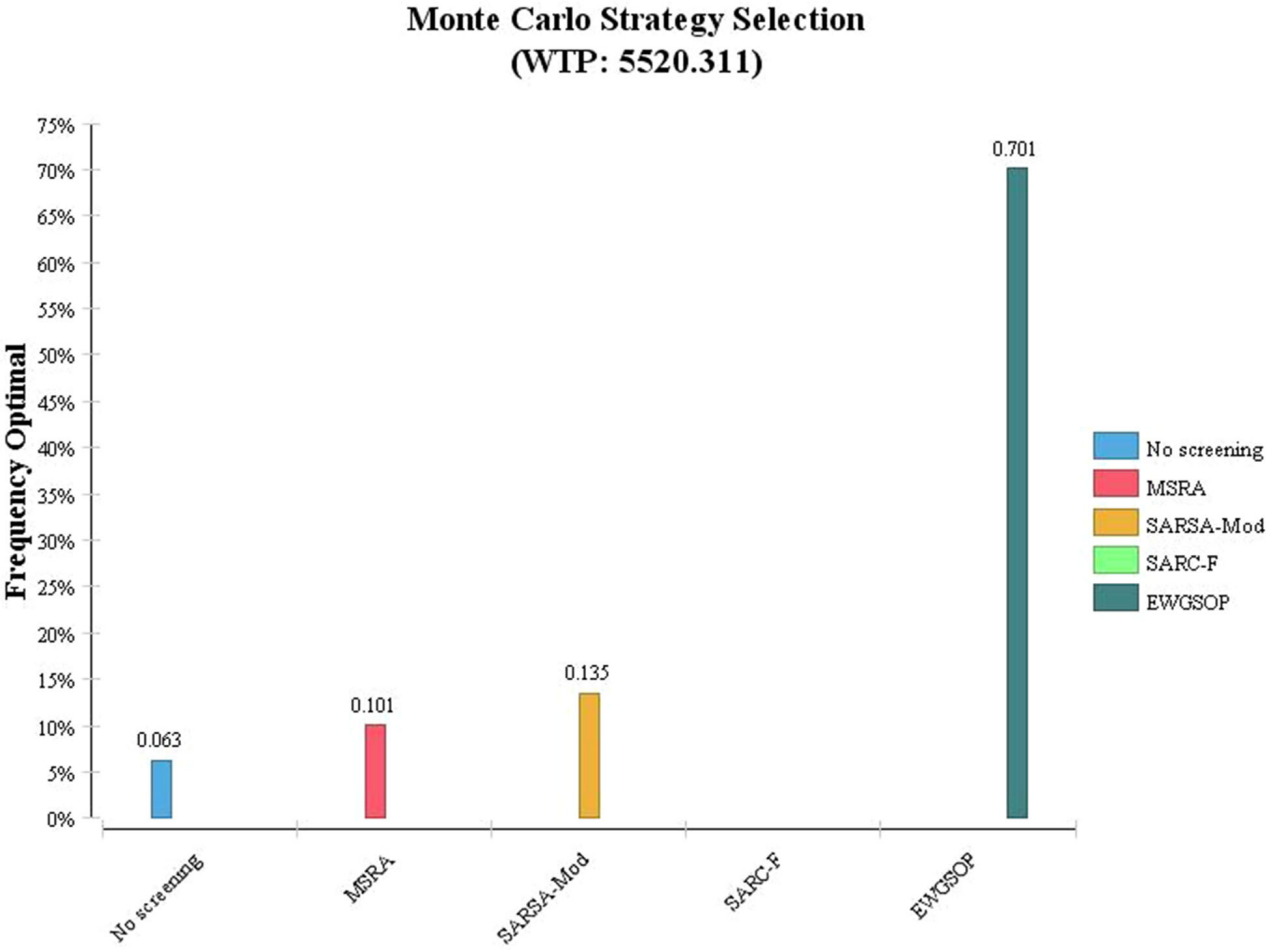
Strategy Selection and Optimization Probability Diagram

The above results can also be deduced from the strategy selection diagram (Fig. 6). As can be seen, considering the cost-effectiveness threshold of $5520.311 and 1000 times of sampling replication in Monte Carlo simulation, the probability of cost-effectiveness of EWGSOP strategy is 70% and the chances of other strategies including SarSA-Mod, no screening and MSRA are 13, 10, and 6%, respectively.

All of the PSA results and Monte Carlo simulation showed that the results of the base case are highly probable robust.

## Discussion

The present study is a full economic evaluation was conducted to compare different strategies of sarcopenia screening. The results of the base case analysis indicated that the EWGSOP strategy was the most cost-effective strategy from a health system perspective. DSA and PSA showed that the base case results were highly robust. It also seems that due to the possibility of identifying patients with high accuracy using this screening method and using timely treatment interventions, higher effectiveness can be achieved than other strategies.

The feasibility of EWGSOP method in all geographical areas of a society is very important. Given that one of the parts in the EWGSOP strategy is to use a DXA bone densitometry device to confirm the diagnosis of sarcopenia [28]. Due to the high budget impact of the DXA, in the short term, it is not possible to use this device in many geographical areas and non-DXA-based methods for sarcopenia screening should be used. The results of this study showed that among the compared strategies, the SarSA-Mod method is the best strategy after EWGSOP in terms of cost effectiveness. With regard to the relatively appropriate sensitivity of this method, the use of simple variables to identify patients, as well as the low budget impact of this strategy, it seems that this method can be used at some levels to identify early at risk sarcopenic individuals. In general, according to the results of the study, the best way to identify early sarcopenic individuals, in areas having DXA devices, is the EWGSOP method, and on the contrary, in areas do not have DXA and areas where the establishment of this device is not cost-effective using simpler screening tools to diagnosis and timely intervene for preventing disease progression is very important, and as the results showed, the SarSA-Mod strategy seems to be the best choice.

With regard to the growing trend of aging in the world, it is important to pay attention to diseases related to elderly ages that timely diagnosis of diseases can assist to reduce overall costs of the health system and also improve the individuals’ quality of life. Sarcopenia, as a disease of elderly age, is also important due to its various possible consequences and significant financial burden, and the timely use of the cost-effectiveness screening strategies for timely intervention will have great importance.

Based on our knowledge, the designed economic model in present study is the first full economic evaluation model to compare sarcopenia screening strategies, which due to all limitations of information, tried to be designed and implemented systematically and step by step based on the best available evidences. Previous studies have focused more on the clinical features of the sarcopenia and in some cases the economic costs of the disease, such as the study of Janssen et al. (2004) and Goates et al. (2019) in the US [33, 52] and the study of Sousa et al. (2016) in Portugal [53].

As mentioned in this model, according to the available evidence, reducing the quality of life, falling, fracture and increased risk of CVDs were considered as clinical outcomes of the disease and other outcomes were ignored due to information constraints. Also in this model, due to lack of access to classified evidence, the division of sarcopenia into pre sarcopenia and severe sarcopenia subgroup was avoided. Another limitation we encountered in designing and implementing the model was the efficacy of treatment and sarcopenia interventions. Since the specific drug has not yet been approved for the treatment and reduction of sarcopenia complications, the efficacy of treatment in this study was based solely on endurance exercises and dietary supplements.

By expanding the evidence and gaining more access to the required parameters and removing study constraints, more complete models can be designed and implemented in the future to identify the most effective strategies.

## Conclusions

The results of the study, as the first economic evaluation of sarcopenia screening, showed that the EWGSOP strategy is more cost-effective than other strategies. Also, Sensitivity analysis showed that the base case results were highly robust.

## Supplementary Information

**Additional file 1: Figure S1.** Prisma Diagram.

**Additional file 2: Table S1.** Comparison between SarSA-Mod, SARC-F and EWGSOP.

## Data Availability

All data generated or analysed during this study are included in this published article [and its supplementary information files]. The datasets used and/or analysed during the current study are available from the corresponding author on reasonable request.

## Abbreviations

CEA: Cost Effectiveness Analysis
SarSA-Mod: Sarcopenia Scoring Assessment Models
EWGSOP: European Working Group on Sarcopenia in Older People
MSRA: Mini Sarcopenia Risk Assessment
AWGS: Asian Working Group for Sarcopenia
QALYs: Quality-Adjusted Life Years
CVDs: Cardiovascular Diseases
BIA: Bio-impedance Analysis
DXA: Dual-energy X-ray absorptiometry
ROC: Receiver Operating Characteristic
ICER: Incremental Cost-Effectiveness Ratio
DSA: Deterministic Sensitivity Analysis
PSA: Probabilistic Sensitivity Analysis
CEAC: Cost Effectiveness Acceptability Curve
