# Supplementary figures and images for: Sarcopenia screening strategies in older people: a cost effectiveness analysis in Iran
Source: BMC Public Health. 2021 May 17;21:926. doi: 10.1186/s12889-021-10511-7 (PMC8127291; doi:10.1186/s12889-021-10511-7)

**Supplementary Information:**


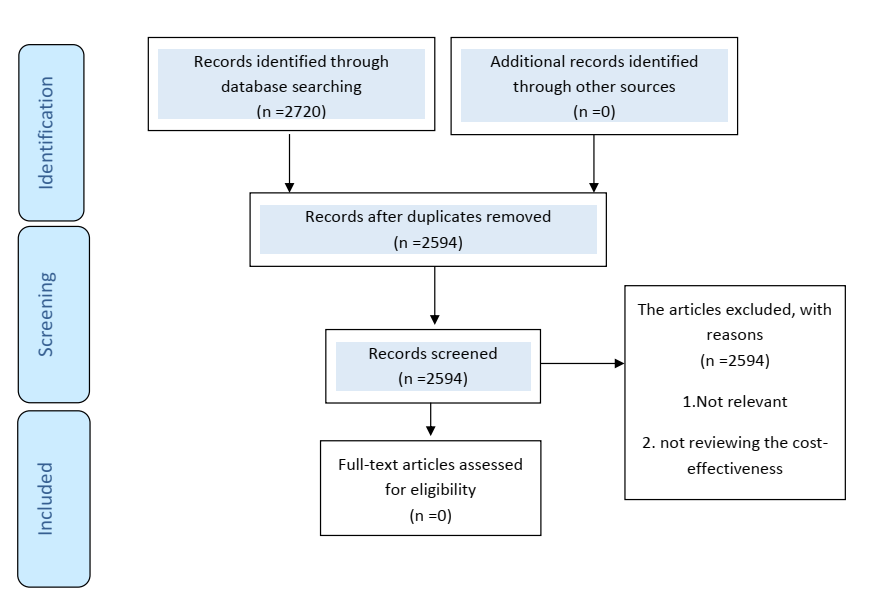


**Fig S1. Prisma Diagram of Systematic Review Results**

Supplement: Supplementary file 1 — Additional file 1: Figure S1. Prisma Diagram. [file 12889_2021_10511_MOESM1_ESM.docx]
