# Supplementary material for: Sarcopenia screening strategies in older people: a cost effectiveness analysis in Iran
Source: BMC Public Health. 2021 May 17;21:926. doi: 10.1186/s12889-021-10511-7 (PMC8127291; doi:10.1186/s12889-021-10511-7)
Supplement: Supplementary file 2 — Additional file 2: Table S1. Comparison between SarSA-Mod, SARC-F and EWGSOP. [file 12889_2021_10511_MOESM2_ESM.docx]

**Supplementary Information:**

| **Table S1^1,2^. Comparison between SarSA-Mod , SARC-F and EWGSOP** | | | | | | |
| --- | --- | --- | --- | --- | --- | --- |
|  | Area under Curve | Sensitivity (%) | Specificity (%) | Positive predictive value (%) | Negative predictive value (%) | Correctly classified (%) |
| **EWGSOP** |  |  |  |  |  |  |
| Men | 0.88 (0.86-0.90) | 100 | 76.2(72.1-79.9) | 68.3 (63.2-73.1) | 100 | 84.3 |
| Women | 0.96(0.94-0.97) | 100 | 91.1 (88.4-93.4) | 82.4 (77.3-86.8) | 100 | 93.7 |
| Total | 0.92 (0.91-0.93) | 100 | 84.0 (81.6-86.2) | 74.3 (70.7-77.7) | 100 | 89.1 |
| **SARC-F** |  |  |  |  |  |  |
| Men | 0.54(0.49-0.58) | 9.5(6.1-13.9) | 97.7(95.9-98.8) | 67.6 (49.5-82.6) | 67.7(64.0-71.2) | 67.7 |
| Women | 0.52(0.46-0.55) | 2.5 (2.0-3.2) | 76.4 (72.5-80.0) | 30.7(24.0 -38.1) | 71.2 (67.3-74.9) | 61.5 |
| Total | 0.52(0.48-0.55) | 16.8 (13.5-20.6) | 86.6(84.3-88.6) | 36. 7(30.1-43.6) | 69.3(66.6-71.8) | 64.5 |
| **SarSA- Mod** |  |  |  |  |  |  |
| Men | 0.89(0.86-0.91) | 81.1 (75.7-85.8) | 83.3 (79.7-86.6) | 71.5(65.8-76.7) | 89.6(86.3-92.3) | 82.6 |
| Women | 0.85 (0.82-0.88) | 80.1(74.1-85.2) | 72.7 (68.6-76.5) | 54.9 (49.2-60.5) | 89.9(86.5-92.5) | 74.9 |
| Total | 0.79 (0.77-0.82) | 80.6 (76.7-84.2) | 77.8(75.0-80.3) | 62. 7(58.3-66.6) | 89.7(87.4-91.6) | 78.7 |
| **SarSA- Mod:** Sarcopenia Scoring Assessment Model, **EWGSOP**: European Working Group on Sarcopenia in Older People  1- Table results is based on assessment of a total of 2211 Iranian adults aged ≥60 years, participating in the stage II of Bushehr Elderly Health program, a population-based prospective cohort study  2-From Gita Shafiee et al. Development and validation of sarcopenia screening model for older people: The Bushehr Elderly Health (BEH) program. | | | | | | |
